# Cannabinoids and monoaminergic system: implications for learning and memory

**DOI:** 10.3389/fnins.2024.1425532

**Published:** 2024-08-14

**Authors:** Sha Zhao, Zhao-Liang Gu, Ya-Nan Yue, Xia Zhang, Yuan Dong

**Affiliations:** ^1^Neuropsychiatry Research Institute, The Affiliated Hospital of Qingdao University, Qingdao, China; ^2^School of Basic Medicine, Qingdao University, Qingdao, China; ^3^Department of Anesthesiology, The Affiliated Hospital of Qingdao University, Qingdao, China

**Keywords:** endocannabinoid, monoamines, learning, memory, dopamine, serotonin

## Abstract

Cannabinoids and the endocannabinoid system (ECS) have been intensively studied for their neuroregulatory roles in the central nervous system (CNS), especially in regulating learning and memory. However, many experimental and clinical studies obtained conflicting results indicating a complex network of interaction underlying the regulation of learning and memory by different cannabinoids and the ECS. The ECS influences neuronal synaptic communications, and therefore may exert different regulation via their different impact on other neurotransmitters. The monoaminergic system includes a variety of neurotransmitters, such as dopamine, norepinephrine, and serotonin, which play important roles in regulating mood, cognition, and reward. The interaction among cannabinoids, ECS and the monoaminergic system has drawn particular attention, especially their contributions to learning and memory. In this review, we summarized the current understanding of how cannabinoids, ECS and the monoaminergic system contribute to the process of learning and memory, and discussed the influences of monoaminergic neurotransmission by cannabinoids and ECS during this process.

## 1 Cannabinoids and the endocannabinoid system

Cannabinoids are first identified in the substances derived from cannabis plants. One of the most prominent contents in plant cannabinoids is Δ-9 tetrahydrocannabinol (THC), which is the main psychoactive substance in cannabis. Up to now, more than 113 different cannabinoids have been identified in cannabis plants (Gulck and Moller, 2020). Cannabis has high heterogeneity, containing more than 600 different chemical components. There are multiple different plant strains containing varying amounts of different plant cannabinoids. Synthetic cannabinoids, a group of chemically synthesized substances that are functionally similar to cannabis, have been intensively studied for pharmacological purposes (Roque-Bravo et al., 2023). Different cannabinoids have been used in the treatment of various diseases, including multiple sclerosis (MS), neuropathic pain, vomiting after chemotherapy, and neuropsychological and cognitive disorders such as depression, anxiety, and sleep disorders. The biological effects of both plant-originated and synthetic cannabinoids are mainly mediated by a class of receptors belonging to the G-protein coupled receptor family: the type-1 and type-2 cannabinoid receptors (CB1R and CB2R). The endogenous cannabinoids (eCBs), anandamide (ANA), and 2-arachidonoylglycerol (2-AG) are some the endogenous lipid ligands of CB1R and CB2R (Busquets-Garcia et al., 2022). The cannabinoid receptors, eCBs, and the enzymes responsible for the biosynthetic and hydrolytic of the eCBs are collectively composed of the endocannabinoid system (ECS) (Lu and Mackie, 2016).

**Figure 1:**
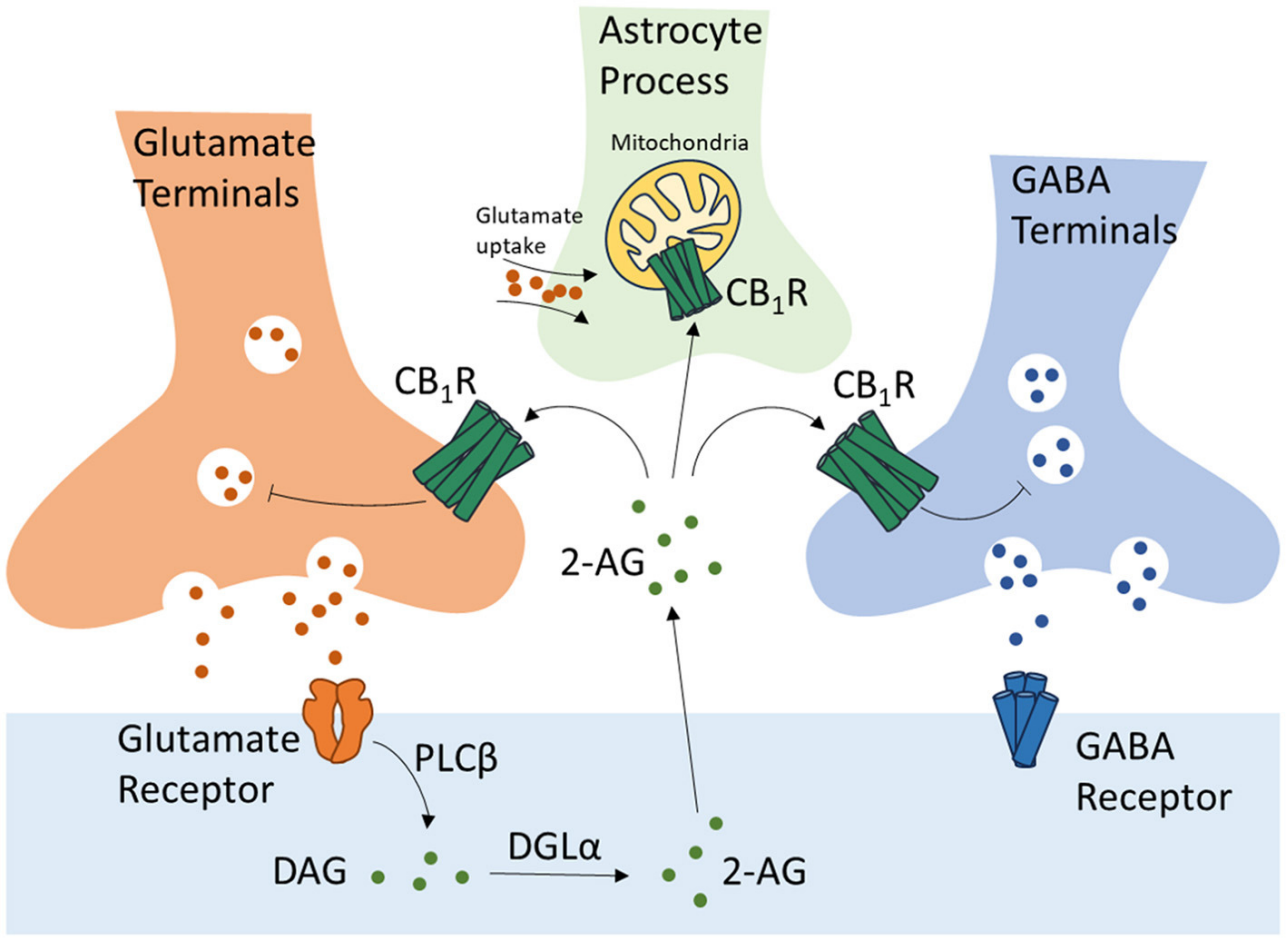
Regulation of glutamate and GABA neurotransmissions, and astrocyte-neuron crosstalk by CB1 receptors. CB1R, Cannabinoid Receptor Type 1; 2-AG, 2-arachidonoylglycerol; DAG, diacylglycerol; DGLα, diacylglycerol lipase-α; PLCβ, phospholipase C-β.

ECS is a widespread modulatory signaling system in the central nervous system (CNS) and plays critical roles in the development of CNS, synaptic plasticity, and the response to endogenous and environmental stimulations. CB1R, located on the presynaptic terminals of GABA and glutamate neurons (Terzian et al., 2014), is activated by eCBs synthesized and released by postsynaptic terminals in response to postsynaptic depolarization. This retrograde regulation of eCBs results in the depolarization-induced suppression of excitation and inhibition (DSE and DSI) causing a transient presynaptic inhibition of neurotransmitter release (Scheyer et al., 2023). CB1R is also expressed by the astrocytes on their membrane and mitochondria (mtCB1R), participating in the regulation of neuronal/synaptic function via astrocyte-neuron crosstalk (Figure 1) (Busquets-Garcia et al., 2018). CB2R, originally identified as a cannabinoid receptor of the peripheral system restricted to immune cells, is now proven to be expressed throughout the CNS. CB2R is expressed at a low level in the microglial cells and astrocytes in a healthy brain. In pathological conditions, such as brain injuries, stroke, and neurodegenerative diseases, the expression of CB2R is highly induced participating in the regulation of inflammation, and therefore has been intensively studied as a treatment target in such diseases (Xin et al., 2020; Yu et al., 2023). The cannabinoids and the ECS are demonstrated to play pivotal roles in the process of learning and memory. However, the exact effects of cannabinoids and the ECS during this process are under debate. In this review, we summarize the recent research advances of how cannabinoids and the ECS affect learning and memory via their interaction with the monoamine neurotransmitters.

## 2 Cannabinoids in learning and memory

How cannabinoids affect learning and memory has been one of the most debated topics for a long time. Many clinical studies reveal no difference in memory, attention, processing speed, and executive function between people with a history of cannabis use and non-user controls (Lyons et al., 2004; Burggren et al., 2018; Panee et al., 2018; Fatjo-Vilas et al., 2020). Meanwhile, other studies support the causal link between cannabis intoxication and learning/memory impairment in a dose-dependent manner (Ranganathan and D'Souza, 2006; Schoeler and Bhattacharyya, 2013; Petker et al., 2019). A meta-analysis based on a large dataset covering more than 43,000 participants reveals the robust association between cannabis use and long-term cognitive impairments (Dellazizzo et al., 2022). A recent randomized trial reveals that administration of THC causes impairments in working memory, increased mind wandering, and decreased metacognitive accuracy (Adam et al., 2020). Consistently, another study demonstrates impairment in memory and processing speed in young adults with positive urine toxicology screen for THC (Petker et al., 2019). THC is considered the main psychoactive component in cannabis causing the acute and adverse effects of cannabis on cognitive functions and memory loss.

In contrast to the clinical studies, treatments using different cannabinoids in rodents demonstrate both impairment or improvement of memory dependent on different stages or tasks of the memory. Also, the type, dosage, and route of administration of the cannabinoid compound, and the age of subjects also seem to influence the outcomes of how cannabinoids affect learning and memory. Chronic low dosage of THC (3 mg/kg per day) for up to 28 days improves memory and spatial learning in old mice (Bilkei-Gorzo et al., 2017). A single low dose of THC (0.002 mg/kg) is demonstrated to improve memory and spatial learning in 24-month-old mice (Sarne et al., 2018). This pro-cognitive effect of THC is likely related to increased volume in the entorhinal cortex, prefrontal cortex, and posterior hippocampus (Sarne et al., 2018). Whereas other components in cannabis, such as cannabidiol (CBD), a non-euphoric component of cannabis, do not induce cognitive impairment. Further research suggests that CBD demonstrates potential anxiolytic and antipsychotic effects, and therefore has been intensively studied for pharmacological purposes (Schoeler and Bhattacharyya, 2013). Studies using synthetic cannabinoids reveal the highly complex regulatory role of ECS in learning and memory. Microinjection of CB1R antagonists (AM251 and SR141716A) into the CA1 region of the hippocampus is found to impair spatial learning in the Morris water maze (MWM) and cause memory acquisition deficit in passive avoidance tasks. Similarly, AM251 injection in basolateral amygdala (BLA) is reported to disrupt the reconsolidation of fear memory. Administration of GABA_A_ receptor antagonist Bicuculline was demonstrated to abolish this effect of AM251, suggesting that this regulation of AM251 was mediated by GABAergic transmission in the BLA (Ratano et al., 2014). Interestingly, Both agonizing and antagonizing of CB1R in CA1 via microinjection of arachidonoylcyclopropylamide (ACPA), a synthetic CB1R-specific agonist, and AM251, respectively, disrupt spatial learning of rats in MWM (Vaseghi et al., 2018). CB1 antagonizing using SR141716 (3 mg/kg) causes increased fear response, meanwhile, CB1 agonizing using CP55940 (50 μg/kg) aggravates the fear response (Llorente-Berzal et al., 2015). Low-dose administration of ACPA at 0.01 mg/kg and AM251 at 50 ng/mouse are reported to cause impairment in memory acquisition (Nasehi et al., 2015). However, in contrast to previous studies, AM251 systematic administration at 1.0 mg/kg is reported to promote recognition memory in rats (Bialuk and Winnicka, 2011). CB1R agonist (WIN55212-2) administration at doses of 1, 3, or 5 mg/kg is found to dose-dependently impair object recognition memory in rats (Baek et al., 2009). However, a lower dose of WIN55212-2 at 0.25 mg/kg is reported to promote the extinction of contextual fear memory and spatial memory in rats (Pamplona et al., 2006). Moreover, the functions of CB1R also seem to exert different impacts on learning and memory under pathological conditions. Both pharmacological stimulation and antagonizing of CB1R demonstrate significant protective effects in rodent models of memory disturbances induced by beta-amyloid (Aβ) peptide brain injection (Mazzola et al., 2003; van der Stelt et al., 2006). This large number of conflicting experimental results collectively reveals the complex nature of how different cannabinoids and the ECS are involved in the regulation of learning and memory.

One of the possible reasons why different cannabinoids and the ECS differently affect learning and memory is that they may interact with different neurotransmitters. Monoamine neurotransmitters are a class of chemical messengers including dopamine, norepinephrine, and 5-hydroxytryptamine (5-HT; serotonin). These neurotransmitters play key roles in the learning and memory process, influencing the formation and maintenance of learning memories by binding to specific receptors that regulate nerve cell activity and synaptic transmission properties. ECS can potentially regulate the monoamine neurotransmission via direct regulation of their release on neuronal terminals, or indirectly via the inhibitory and excitatory projections on the neurons that are able to release monoamine neurotransmitters. As CB1R is believed to be differently expressed in different neuronal subpopulations, with a higher level on GABAergic interneurons and a lower level on glutamatergic terminals (Marsicano and Lutz, 1999), their impact on monoamine neurotransmission and the process of learning and memory is therefore complex. In the following parts, we summarize the current understanding of how cannabinoids and the ECS interact with the monoaminergic system, and how this interaction is involved in the regulation of learning and memory.

## 3 The interaction between endogenous cannabinoids and monoamine neurotransmitters in learning and memory

### 3.1 Dopaminergic neurotransmission

#### 3.1.1 Dopaminergic neurotransmission in learning and memory

The dopamine system is related to many different aspects of normal brain functions, including learning and memory (Grella and Donaldson, 2024), emotions (Wakeford et al., 2024), and cognitions (Sun et al., 2024). Interestingly, an inverted U-shaped curve has been proposed between dopamine signaling and cognitive performance (Weber et al., 2022). Specifically, optimal levels of dopamine and D1 dopamine receptor are required for best cognitive function, excessively high or low dopamine signaling impairs cognitive performance. Consistently, both agonizing and antagonizing of D1 dopamine receptor in the BLA are found to impair context-dependent fear learning (Nasehi et al., 2016). This dose-dependent effect of dopamine on cognitions indicates its prominent regulatory role during this process. Indeed, many studies indicate that the dopamine system plays a crucial role in various cognitive functions by regulating blood oxygen level-dependent (BOLD) signals (Salami et al., 2019). Anatomically, the dopamine-expressing neurons are widely distributed throughout the CNS. Meanwhile, intense dopaminergic projections connect the striatum, hippocampus, prefrontal cortex and the limbic system. This dopaminergic network plays an important role in the formation and processing of associative memory. The dopamine system in the amygdala is largely involved in the formation of fear-regulated memory (Ritger et al., 2024). In the hippocampus, dopamine plays an important role in the transition of CA1-CA3 synapses from early long-term potentiation (E-LTP) to late long-term potentiation (L-LTP) (Frey et al., 1990). Moreover, D1/D5 dopamine receptor agonists enhance memory function by mimicking the neurophysiological effects of dopamine in learning and memory (Hersi et al., 1995). However, there is limited research on the function of D2-like dopamine receptors, and their role in cognitive function has not been fully elucidated. Previous studies have shown that PFC infusion of the D2 agonist 2-(n-phenylethyl-n-propyl)-amino-5-hydroxytetrahydroalin hydrochloride (PPHT), dose-dependently disrupts spatial working memory in rats undergoing delayed U-maze tasks (Druzin et al., 2000). Quipilol (D2 receptor agonist) has a systemic dose-dependent effect on spatial working memory in delayed response tasks in young adult monkeys, mainly manifested as a protective effect at moderate doses and memory damage at high or low doses. It is worth noting that dopamine exhibits a high affinity for D4 receptors, which are highly expressed in brain regions related to learning and memory (Alachkar et al., 2024). In addition, the dopaminergic system is also involved in processing information related to rewards. In the ventral tegmental area (VTA) and substantia nigra, dopaminergic neurons are able to track reward prediction errors and emit signals with typical features of learning positive reinforcement signals (Yang et al., 2024). Although the dopaminergic system is considered a global neural regulatory system, it can provide precise temporal information for specific target structures, affecting many cognitive functions.

#### 3.1.2 ECS influences dopaminergic neurotransmission

The ECS plays an important role in regulating reward-related changes in dopamine levels in the nucleus accumbens (NAc) and other brain regions associated with addiction (Honeywell et al., 2024). THC or WIN55212-2 administration increases dopamine levels in primates and rodents, and exposure to THC significantly increases early adult opioid self-administration and enhances the release of midline dopamine in rats (Justinova et al., 2013; Terzian et al., 2014). Systemic injection of cannabinoids (THC or WIN55212-2) enhances the excitability of PFC neurons toward VTA dopaminergic input and indirectly increases the excitability of VTA dopaminergic neurons by inhibiting the GABAergic projection to VTA (Pistis et al., 2001). The activation of CB1R in PFC changes the activation of downstream dopaminergic neurons in VTA in a biphasic and dose-dependent manner, leading to disturbances in emotional memory processing (Pistis et al., 2001). Intra-BLA injection of D1 receptor agonist SKF38393 (1μg per mouse) or D2 receptor agonist quinpirole (0.1 μg per mouse) is demonstrated to rescue the ACPA-induced memory acquisition impairment. Those regulatory roles of cannabinoids in dopaminergic transmission and dopamine-related behavior appear to be indirect, and may be exerted via the influences of CB1R on GABAergic or glutamatergic terminals projecting on dopaminergic neurons, as CB1R appears to be present in both glutamatergic projection and GABAergic inhibitory neurons (Nasehi et al., 2016). Expression of CB1R is also reported on neurons expressing D1 dopamine receptors (Terzian et al., 2011; Micale et al., 2017). Conditional knockout of CB1R on D1 dopamine receptor-expressing cells causes weak- to moderate anxiety-like behaviors and significantly elevated contextual and auditory-cued fear (Terzian et al., 2011), indicating the possible influences of dopamine downstream regulations by ECS.

One recent study reveals the expression of CB1R on dopamine neurons in VTA related to rewarding. Conditional knockout of CB1R on dopamine neurons abolishes the inhibitory effects of THC or arachidonyl-2′-chloroethylamide (ACEA, a highly selective cannabinoid CB1 receptor agonist) administration on rewarding as revealed by dose-dependently reduced optical intracranial self-stimulation, indicating the direct regulation of CB1R on dopamine release and related behaviors (Han et al., 2023). However, the mechanism of how ECS interacts with dopamine neurotransmission, especially its influences on the process of learning and memory, is not fully understood. Acute systematic injections of THC or WIN55212-2 are reported to increase dopamine accumulation in the hippocampus, indicating a possible regulation of learning and memory via dopaminergic neurotransmission by cannabinoids (Moranta et al., 2004), and therefore worth more in-depth studies.

Other than influencing dopamine levels, cannabinoids have also been suggested to modulate the transcription of dopamine receptors. Perinatal THC exposure in rats is associated with schizophrenia in adulthood, as revealed by social withdrawal and cognitive impairments (Di Bartolomeo et al., 2021, 2023). Those abnormalities are due to increased transcriptional levels of CB1R and D2/D3 dopamine receptors in the PFC (Stark et al., 2020; Di Bartolomeo et al., 2021, 2023). Interestingly, THC treatment in adults is demonstrated to rescue the cognitive deficit in prenatal methylazoxymethanol acetate (MAM) exposure-induced schizophrenia, rather than perinatal THC exposure-induced schizophrenia in rats, by modulating the expression of D2/D3 dopamine receptors (Di Bartolomeo et al., 2023). Meanwhile, CDB treatment is reported to reverse the prenatal MAM exposed-induced schizophrenia by changing the expression of D3 dopamine receptor (Stark et al., 2020). Collectively, those studies not only indicate the important roles played by cannabinoid-dopamine interaction in schizophrenia, but also suggest the critical role of their interactions during ontogenetic development. As ECS has been reported to play critical roles in regulating the neurogenic processes during ontogenetic development (Gomes et al., 2020), how this ontogenetic variation of ECS influences dopamine neurotransmission and contributes to the processes of learning and memory is particularly interesting.

### 3.2 5-HT neurotransmission

#### 3.2.1 5-HT neurotransmission in learning and memory

Eric Kandel demonstrated the decisive contribution of 5-HT to memory formation in the 1970s. 5-HT induces the level of 3′, 5′-cyclic adenosine monophosphate (cAMP) in sensory neurons of California sea hare (*Aplysia californica*) (Cedar and Schwartz, 1972). This process activates cAMP-dependent protein kinases, which promote synaptic transmission in sensitization and induce synaptic strength-dependent LTP of protein synthesis after repeated stimulation (Longo et al., 1990). Since then, the contribution of 5-HT in the memory process has been intensively studied and proposed as a promising target for the treatment of memory-related disorders. 5-HT receptors contain seven different subtypes, including 5-HT1 receptor to 5-HT7 receptor (Sharp and Barnes, 2020). Except for 5-HT3 receptor, which belongs to the ligand-gated ion channel belongs to the cys-loop channel family, all of the rest are G-protein-coupled receptors (GPCRs), which couple to at least 13 types of G proteins, and are involved in a multitude of physiological and pathological processes (Noda et al., 2004; Hannon and Hoyer, 2008).

5-HT receptors are heterogeneously distributed in the brain and activate different downstream signaling pathways according to their coupled G proteins. However, the exact role of 5-HT in learning and memory regulation is not fully understood, partially due to the complex regulatory role carried out by distinct subtypes of 5-HT receptors. For example, stimulation of 5-HT1A receptor, coupled with inhibitory G proteins (G_i_/G_o_), generally demonstrates learning impairment effects (Ogren et al., 2008). In contrast, in another study, blocking of 5-HT1 receptors is reported to enhance the hippocampal activities, and therefore can be targeted as a potential therapeutic option for depression or diseases related to memory deficits (Jahreis et al., 2023). Administration of 5-HT1B receptor agonist anpirtoline via subcutaneous injection produce dose-dependent impairment of spatial learning in rats, and 5-HT1B receptor selective antagonist NAS-181 fully rescues the impairment of learning caused by anpirtoline, indicating the important role of 5-HT1B receptor in the processes of learning and memory (Ahlander-Luttgen et al., 2003). 5-HT2A receptor is reported to influence memory in human (de Quervain et al., 2003). 5-HT2A receptor, a G_q/11_-coupled G protein, is able to form heterodimer with dopamine D_2_ receptors and mGluR2 receptor (Gonzalez-Maeso et al., 2008; Lukasiewicz et al., 2010), and therefore participates in the process of learning and memory. 5-HT2A receptor is also demonstrated to directly interact with NMDA receptor. Postsynaptic 5-HT2A receptor regulates object memory consolidation via modulating NMDAR-mediated synaptic plasticity (Peddie et al., 2008). Therapeutic approaches targeting 5-HT2A receptor has been indicated as a promising way for the treatment of learning and memory impairment associated with neurodegenerative diseases, and also a possible approach to regulate addiction (Zhang and Stackman, 2015). 5-HT3 receptor is not coupled with G proteins. Systemic injection of 5-HT3 receptor antagonist is reported to promote the induction of LTP and enhance the retention of memory (Staubli and Xu, 1995). Mice with 5-HT3 receptor knockout demonstrate impaired fear extinction (Kondo et al., 2013). 5-HT4 receptor, coupled with stimulatory G proteins (G_s_), is also reported to promote memory acquisition and consolidation in both human and mouse (Teixeira et al., 2018; Murphy et al., 2020). The exact function of 5-HT5 receptor is not clear. It is reported to couple with G_i_/G_o_ or G_s_. In learning and memory, 5-HT5A receptor is demonstrated to promote memory, as pharmacological blockade of 5-HT5A receptor impairs both short-term and long-term memory, while 5-HT5A receptor stimulation enhances memory (Gonzalez et al., 2013). 5-HT6 receptor, although predicted to couple with G_s_ (Hannon and Hoyer, 2008), is found to negatively influence learning and memory. Specifically, using selective 5-HT6 receptor antagonists is found to promote learning and memory via increased cholinergic neurotransmission (Meneses et al., 2007), while pharmacological stimulation of 5-HT6 receptor is reported to impair both short- and long-term memories (Meneses et al., 2008). 5-HT7 receptors are reported to couple with G_s_ in human (Hannon and Hoyer, 2008). 5-HT7 receptors agonizing using AS19 is reported to impair short-term memory, and this impairing effect is rescued by selective 5-HT7 receptor antagonist SB-269970, but not by elective 5-HT1A antagonist WAY 100635 (Meneses et al., 2008). Interestingly, 8-Hydroxy-2-(dipropylamino) tetralin (8-OH-DPAT), an agonist for both 5-HT1A and 5-HT7 receptors, is demonstrated to cause contextual learning impairment. However, its impact on learning through different receptors appears to be different. It seems that the contextual learning impairment caused by 8-OH-DPAT is due to its stimulation of postsynaptic 5-HT1A receptors. Meanwhile, use of 5-HT7 receptor antagonist exaggerated contextual learning impairment caused by 8-OH-DPAT, indicating that the activation of 5-HT7 receptor by 8-OH-DPAT actually counteracts the 5-HT1A receptor-mediated impairments caused by 8-OH-DPAT (Eriksson et al., 2008). Collectively, 5-HT neurotransmission is a major regulator of neuronal processes related to learning and memory. However, due to the complexity of 5-HT receptors and their distinct functions, influences of 5-HT on memory is complex and worth more in-depth investigations.

#### 3.2.2 5-HT neurotransmission regulated by ECS

ECS has been reported to influence 5-HT neurotransmission via regulating 5-HT release and the expression of 5-HT receptors. Systemic THC administration is found to increase 5-HT level in rats (Segawa and Takeuchi, 1976). Specifically, systemic injection of WIN55212-2 is found to CB1R-dependently promotes the spontaneous firing of 5-HT neuron in dorsal raphe nucleus (DRN) (Bambico et al., 2007). CB1R systematical agonizing using WIN55212-2 and CP55940 causes increased 5-HT efflux in NAc, one of the projection areas of DRN, in rats (Tao and Ma, 2012). This effect is found to mediated by the CB1R-dependent inhibition of GABAergic interneurons in the DRN (Tao and Ma, 2012). Deactivation of fatty acid amide hydrolase (FAAH), the endocannabinoid hydrolase, is also reported to promote the activity of 5-HT neuron in DRN (Gobbi et al., 2005; Bambico et al., 2010). However, reduced or unchanged 5-HT level has also reported in other brain regions. THC administration is found to induce spatial memory impairment via reduced 5-HT release in the medial PFC and hippocampus (Egashira et al., 2002). Reduced level of 5-HT is also reported in NAc after THC administration (Sano et al., 2008). WIN55212-2 is found to reduce the release of 5-HT in frontocortical (Ferreira et al., 2012).

**Figure 2:**
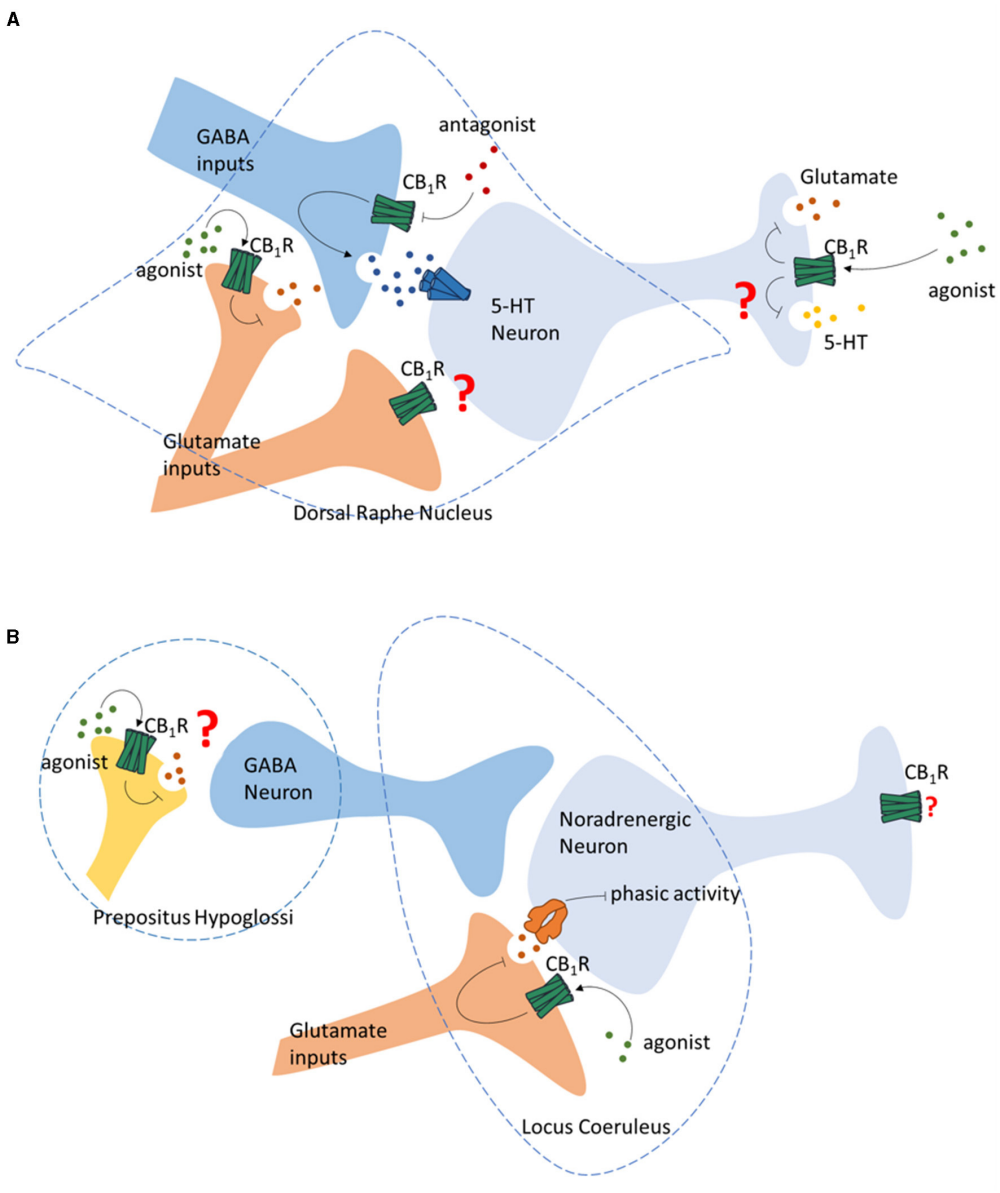
Regulation of monoamine neurotransmission by CB1R. **(A)** Regulation of the 5-HT neurons in Dorsal Raphe Nucleus by GABA and glutamate terminals by presynaptic CB1R, and the regulation of glutamate and 5-HT release of 5-HT neurons by CB1R. **(B)** Regulation of the noradrenergic neurons in Locus Coeruleus by ECS.

Electrophysiological studies *in vitro* demonstrate that the CB1R agonists infusion produce no significant effect on the neuronal activity of 5-HT neuron in DRN. Meanwhile, CB1R antagonist infusion suppresses the 5-HT neuron firing in DRN (Mendiguren and Pineda, 2009), suggesting an eCBs activating effect of the 5-HT neuron (Figure 2A). The influence of 5-HT neurotransmission also seems to be indirect via the inhibitory projections on the 5-HT neurons in DRN, as revealed by the finding that the inhibitory effect of CB1R antagonizing is abolished by GABA_A_ receptor antagonist (Mendiguren and Pineda, 2009) (Figure 2A). CB1R is also reported to locate on the glutamate terminals in the DRN originated from PFC (Marsicano and Lutz, 1999) (Figure 2A). PFC lesion abolishes the 5-HT stimulatory effect induced by systemic injection of WIN55212-2, indicating the regulation of 5-HT release DRN by ECS via glutamate terminals (Bambico et al., 2007). However, the exact function of glutamate terminals regulating 5-HT neuron in DRN is not conclusive (Haj-Dahmane and Shen, 2011; Mendiguren et al., 2018; Peters et al., 2021).

The DRN of the midbrain is a major source of 5-HT in the CNS, including the hippocampus, which is the brain region related to learning and memory. Co-expression of tryptophan hydroxylase 2 (TPH2), a 5-HT neuron marker, and CB1R mRNA is reported in the DRN in rodents (Haring et al., 2007). CB1R is also reported to locate on the axon terminals of 5-HT neurons (Carvalho et al., 2010). Similarly, single-cell transcriptome analysis suggests that in the mouse DRN, the CB1R is often co-expressed on 5-HT neurons. This group of neurons also release glutamate, indicating a possible regulation of 5-HT release by the ECS at their terminals (Ren et al., 2019; Wang et al., 2019; Okaty et al., 2020) (Figure 2A).

Other than influencing 5-HT release, the ECS is reported to alter the expression of 5-HT receptors in the CNS. CB1R knockout or pharmacological blockade is reported to desensitize 5-HT1A receptor, suppress 5-HT2C receptor expression in DRN, NAc and the paraventricular nucleus of the hypothalamus (PVN), and promote the expression of 5-HT2C receptor in CA3 of the ventral hippocampus (Aso et al., 2009).

Overall, CB1R stimulation is believed to increase 5-HT levels in brain regions such as DRN and NAc. However, the exact influence seems dependent on the type, concentration, and route of cannabinoids that been used, and the brain region that the 5-HT been tested. Indeed, acute THC and WIN55212-2 systematic administrations are previously demonstrated to reduce the hippocampal level of 5-hydroxytryptophan (5-HTP), which is a precursor for 5-HT (Moranta et al., 2004). Differences of how cannabinoids influence 5-HT in the brain is also probably caused by the indirect effects of eCBs at GABA neurons, suggesting that the ECS may produce different effect on neuron activities depending on different regulation of excitatory/inhibitory neuronal circuit (Peters et al., 2021). Moreover, as summarized in the previous section, functions of different 5-HT receptors are expected to be distinct, especially in the regulation of learning and memory. How altered 5-HT level caused by different cannabinoids and ECS contribute to learning and memory via distinct 5-HT receptors is unknown. These distinct 5-HT neurotransmission regulations by cannabinoids and ECS could partially explain the complexity of how cannabinoids and ECS impact the process of learning and memory. For this reason, further investigations of how the use of different cannabinoids and alternations in ECS affect the neurocircuits related to learning and memory via their impact on 5-HT neurotransmission and different 5-HT receptors are critically important.

### 3.3 Noradrenergic neurotransmissions

#### 3.3.1 Noradrenergic neurotransmission in learning and memory

Norepinephrine is a neurotransmitter that plays a key role in the brain and is involved in a wide range of physiological activities, including attention, emotion regulation, and stress response (Sara, 2009; Bahtiyar et al., 2020). Animal studies have demonstrated the regulatory role of the noradrenergic system in memory consolidation (Balbinot and Haubrich, 2023). Norepinephrine acts on both pre- and post-synaptic adrenergic receptors in the brain and participates in the regulation of learning and memory mainly by the modulation of synaptic plasticity (Tully and Bolshakov, 2010). Norepinephrine is found to reduce the threshold for LTP in the hippocampus via the phosphorylation of the GluR subunit of AMPARs and therefore enhances learning and memory (Hu et al., 2007). Noradrenergic neurons are mainly distributed in the brainstem, namely the locus coeruleus (LC) and solitary tract nucleus (NTS) (Szabadi, 2013). Specifically, LC is reported as the largest noradrenergic nucleus in the brain, and the only source of norepinephrine in the forebrain and the hippocampus (O'Dell et al., 2015; Galgani et al., 2023). Activation of the LC-norepinephrine system plays an important regulatory role in learning and memory (Giustino and Maren, 2018). The noradrenergic projection from LC to the hippocampus is reported to promote memory consolidation by releasing norepinephrine, which induces a long-lasting enhancement of synaptic transmission effects (Hansen, 2017). Significantly reduced levels of norepinephrine and its metabolites are observed in the LC-hippocampus-cortex system of aged rats, which may be associated with their increased phobias as well as deficits in spatial learning and memory, confirming that the efficiency of noradrenergic neurotransmissions affects learning and memory (Collier et al., 2004). Bilateral inactivation of LC is reported to impair the acquisition of memory (Khakpour-Taleghani et al., 2009), while LC activation increases norepinephrine levels in DG and CA1, which leads to enhanced LTP and long-term depression (LTD), and subsequently promotes the encoding of memory (Katsuki et al., 1997; Lemon et al., 2009). Norepinephrine acts on β-adrenergic receptors (β-ARs) leading to hippocampal LTD and promoting LTD-related memory processing (Hagena et al., 2016). In the hippocampus, dentate gyrus (DG) contains the highest concentration of receptors and the highest fiber density projected from LC, resulting in the highest levels of norepinephrine release (Chowdhury et al., 2022). Moreover, nisoxetine (norepinephrine reuptake inhibitor) or idazosin (α2 adrenergic receptor antagonist) administration is reported to enhance LTP, while clonidine (α2 adrenergic receptor agonist) impairs LTP (Garrido Zinn et al., 2018; Nguyen and Gelinas, 2018; Saggu et al., 2023). Collectively, norepinephrine enhances synaptic plasticity in the hippocampus through the activation of β-ARs, and in particular plays an important role in the formation and stabilization of LTP.

#### 3.3.2 Regulation of noradrenergic neurotransmissions by ECS

Early anatomical studies using autoradiography reveal moderate CB1R mRNA in the primary noradrenergic nucleus LC and NTS (Scavone et al., 2010; Navia-Paldanius et al., 2015). The characterization of the distribution of CB1R in LC indicates that CB1R is localized within the somatic dendritic spectrum and axonal terminals. The neurochemical characterization of LC neurons indicates that some CB1R-positive neurons are noradrenergic (Wyrofsky et al., 2019), indicating potential interaction between the ECS and noradrenergic neurotransmissions. Indeed, systemic administration of synthetic cannabinoids (WIN55212-2 and CP 55940) and THC has been shown to increase the spontaneous firing rate of neurons in LC in rodents (Mendiguren and Pineda, 2006; Muntoni et al., 2006). Similarly, increased LC cell activities are observed in inhibiting the degradation of eCBs using FAAH inhibitor URB597 (Gobbi et al., 2005). Consistently, systemic administration of WIN55212-2 and CP55940 has been shown to increase c-fos expression in LC noradrenergic neurons and norepinephrine release in the downstream nucleus (Patel and Hillard, 2003; Oropeza et al., 2005). This increased LC noradrenergic neuron activities induced by CB1R stimulation are also associated with elevated tyrosine hydroxylase (TH) expression and NA synthesis in the LC and the efferent brain areas of LC (Moranta et al., 2004; Page et al., 2007). Systemically administration of rimonabant, a CB1R antagonist, is reported to decrease the firing rate of noradrenergic neurons in LC (Muntoni et al., 2006). However, activation of CB1R by LC local administration of URB597 is not able to increase c-fos expression in LC noradrenergic neurons (Murillo-Rodriguez et al., 2007). Further study reveals that the stimulation of LC neuron activity by systemic cannabinoid administration may be due to disinhibition of the LC noradrenergic neurons via the activation of CB1R in prepositus hypoglossi (PrH), rather than activation of CB1R in LC (Mendiguren and Pineda, 2006; Muntoni et al., 2006) (Figure 2B). Other than regulating the GABAergic projection to LC, CB1R is also reported to regulate the glutamatergic projection to LC. *In vitro* studies reveal that cannabinoids can decrease the phasic activity of LC cells by reducing glutamate release via a local microcircuit (Mendiguren et al., 2018) (Figure 2B).

Collectively, those studies have suggested the general stimulation of noradrenergic neurons in LC and efferent areas by activation of CB1R. However, how this interaction between cannabinoids and norepinephrine implicates the process of learning and memory is currently unknown. In the hippocampus, as one of the most potent efferent brain regions of noradrenergic neurons in LC, changes in noradrenaline release or synthesis by different cannabinoids may provide some insights. Specifically, acute and chronic (5 days) systematic administration of WIN55212-2 at doses ranging from 2 to 8 mg/kg are reported to reduce noradrenaline content in the hippocampus of rats (Moranta et al., 2004, 2009). Similarly, THC systematic administration (5, 10, 20 mg/kg) is also reported to reduce the noradrenaline content in the hippocampus of rats (Moranta et al., 2004). CB1R systematic antagonizing using rimonabant (3 mg/kg) is found to increase noradrenaline release in the hippocampus (Tzavara et al., 2001). Consistently, *in vitro* studies suggest reduced noradrenaline release in the hippocampus of human and guinea pig induced by WIN55212-2 or CP55940 (Schlicker et al., 1997; Kathmann et al., 1999). Together with the important roles played by the hippocampal noradrenergic neurotransmissions in learning and memory, it seems that cannabinoids may exert potential influences on learning and memory via its regulation of noradrenergic neurotransmissions. Further research regarding this topic is, therefore, necessary to identify the mechanism and neurocircuit underlying the regulation of noradrenergic neurotransmissions by cannabinoids and ECS in learning and memory.

## 4 Conclusion remarks and future perspectives

The use of cannabinoids and alternation in the ECS has been suggested to intensively influence learning and memory. Many conflicting results have been obtained indicating a complex regulating network behind this important regulation. The interaction between ECS and monoaminergic system may participate in the regulation of learning and memory. However, the exact mechanism and neurocircuit of this interaction and its role in learning and memory is not clear. In this review, we summarize and discuss the current understanding of how cannabinoids and the ECS interact with the monoaminergic system, and how this interaction is potentially involved in the regulation of learning and memory. However, there are still many issues to be addressed in future investigations:

1. In most studies, cannabinoids are delivered via systematic administration, which affects the ECS in both CNS and peripheral systems. This may be the reason why the effect of certain cannabinoids varies depending on the dosage and route of administration. In future studies, brain region-specific and neuronal cell type-specific studies are necessary for a better understanding of how cannabinoids and ECS are involved in the regulation of learning and memory.
2. The monoamine system of the brain has important features of automatic and cross-regulation. The regulatory effects of cannabinoids and the ECS are able to fine-tun monoamine system. For this reason, more in-depth studies investigating the complex regulation between ECS and monoamine system, using new genetic, pharmacological, and viral approaches, are necessary to reveal the neuro-modulatory process in learning and memory.

## Author contributions

SZ: Writing – original draft. Z-LG: Writing – original draft. Y-NY: Writing – original draft. XZ: Conceptualization, Funding acquisition, Writing – review & editing. YD: Conceptualization, Methodology, Writing – review & editing.
